# Dynamics of fecal microbial communities in children with diarrhea of unknown etiology and genomic analysis of associated *Streptococcus lutetiensis*

**DOI:** 10.1186/1471-2180-13-141

**Published:** 2013-06-19

**Authors:** Dong Jin, Chen Chen, Lianqing Li, Shan Lu, Zhenjun Li, Zhemin Zhou, Huaiqi Jing, Yanmei Xu, Pengcheng Du, Haiyin Wang, Yanwen Xiong, Han Zheng, Xuemei Bai, Hui Sun, Lei Wang, Changyun Ye, Marcelo Gottschalk, Jianguo Xu

**Affiliations:** 1 State Key Laboratory for Infectious Disease Prevention and Control, and National Institute for Communicable Disease Control and Prevention, Chinese Center for Disease Control and Prevention, Beijing, 102206, China; 2 Molecular Microbiology Laboratory, ShanXi Children’s Hospital, TaiYuan, 030013, China; 3 College of Life Sciences, Nankai University, Tianjin, China; 4 Groupe de Recherche sur les Maladies Infectieuses du Porc, Faculté de médecine vétérinaire, Université de Montréal, Québec, Canada

**Keywords:** Microbial communities, 16S rRNA gene analysis, *Streptococcus lutetiensis*, Genome analysis, Pathogenic island

## Abstract

**Background:**

The sequences of the 16S rRNA genes extracted from fecal samples provide insights into the dynamics of fecal microflora. This potentially gives valuable etiological information for patients whose conditions have been ascribed to unknown pathogens, which cannot be accomplished using routine culture methods. We studied 33 children with diarrhea who were admitted to the Children’s Hospital in Shanxi Province during 2006.

**Results:**

Nineteen of 33 children with diarrhea could not be etiologically diagnosed by routine culture and polymerase chain reaction methods. Eleven of 19 children with diarrhea of unknown etiology had *Streptococcus* as the most dominant fecal bacterial genus at admission. Eight of nine children whom three consecutive fecal samples were collected had *Streptococcus* as the dominant fecal bacterial genus, including three in the *Streptococcus bovis* group and three *Streptococcus* sp*.*, which was reduced during and after recovery. We isolated strains that were possibly from the *S. bovis* group from feces sampled at admission, which were then identified as *Streptococcus lutetiensis* from one child and *Streptococcus gallolyticus* subsp. *pasteurianus* from two children. We sequenced the genome of *S. lutetiensis* and identified five antibiotic islands, two pathogenicity islands, and five unique genomic islands. The identified virulence genes included hemolytic toxin *cylZ* of *Streptococcus agalactiae* and sortase associated with colonization of pathogenic streptococci.

**Conclusions:**

We identified *S. lutetiensis* and *S*. *gallolyticus* subsp. *pasteurianus* from children with diarrhea of unknown etiology, and found pathogenic islands and virulence genes in the genome of *S. lutetiensis.*

## Background

In the developing world, every child under 5 years of age experiences approximately three episodes per year of diarrhea [1]. Although more than 200 viral, bacterial, and parasitic causes of diarrhea have been identified to date, only a few etiological agents cause the vast majority of diarrheal diseases in children in the developing world. These include rotavirus, diarrheagenic *Escherichia coli*, *Campylobacter jejuni*, *Shigella* spp., non-typhoidal *Salmonella*, *Giardia lamblia*, *Cryptosporidium* spp. and *Entamoeba histolytica*[2]. Unfortunately, a large proportion of cases of diarrheal disease are of unknown etiology. There are many reasons for this problem, including fragility of causative agents, exacting growth requirements, and lack of recognition of some organisms as enteric pathogens.

Here, we used the previously described strategy of 16S rRNA gene polymerase chain reaction (PCR) and sequencing technology [3] to analyze quantitatively the densities of different bacterial species in fecal samples of patients with diarrhea of unknown etiology at different times relative to hospital admission, and analyzed the features of the dominant species.

## Methods

### Study design

Children with diarrhea without antibiotic treatment who were admitted to the Children’s Hospital, Shanxi Province, China from August 17 to 30, 2006 were screened for enteric pathogens, including *Shigella*, *Salmonella*, enterotoxigenic *E. coli*, enteroinvasive *E. coli* (EIEC), enteropathogenic *E. coli* (EPEC), Shiga-toxin-producing *E. coli*, enteroaggregative adherence *E. coli* (EAEC), and common diarrhea viruses, including group A rotavirus, human calicivirus (HuCV), enteric adenovirus (Adv) and human astrovirus (HAstV). The targeted virulence genes of enteric bacterial pathogens included heat-labile (LT), heat-stable (ST) enterotoxins, Shiga-like toxin (SLT), bundle forming pili (*bfpA*), enteric attaching and effacing locus (*eaeA*), EAEC specific probe, and the genes encoding invasive plasmid antigens (*ipaBCD*) [4-7]. The group A rotaviruses were detected by commercially available enzyme-linked immunosorbent assay (ELISA) according to the manufacturer’s instructions (Oxoid, Basingstoke, UK). Total viral DNA and RNA were extracted from fecal specimens prepared in phosphate-buffered saline at 10%(wt/vol) using the QIAamp MinElute Virus Spin Kit (Qiagen, Hilden, Germany) according to the manufacturer’s recommendations. HuCV, enteric Adv and HAstV were detected by PCR as described previously [8-10]. *G. lamblia* and *Ent. histolytica* were detected using direct microscopy with a saline preparation of the specimen. The clinical history and physiological findings of each patient were documented on standardized case report forms. Fecal samples from five healthy and five hospitalized children at the same location but with no apparent diarrhea were analyzed as controls. Libraries of the 16S rRNA gene were constructed for each fecal sample, with a minimum size of 100 analyzable sequences [11].

### Analyzing dominant fecal bacterial species by 16S rRNA gene sequence technology

All fecal samples were collected in triplicate; one for timely isolation and detection of the enteric pathogens; one stored at −20°C for 16S rRNA sequence analysis; and one stored in 20% glycerol at −80°C for isolation of the putative pathogens suggested by the 16S rRNA gene analysis.

The DNA was extracted from a 200-mg fecal sample, which was measured and adjusted to 100 ng/μl of each sample for PCR. The universal eubacterial primers 27 F-519R (5’-agagtttgatcmtggctcag-3’ and 5’-gwattaccgcggckgctg-3’) were used to amplify a 500-bp region of the 16S rRNA gene. LaTaq polymerase (TaKaRa, Dalian, China) was used for PCR under the following conditions: 95°C for 5 min, followed by 20 cycles of: 95°C for 30 s, 52°C for 30 s, and 72°C for 1 min; and a final elongation step at 72°C for 10 min.

The PCR products were extracted from sliced gels and cloned into the pGEM^R^-T Easy Vector System (Promega, Madison, WI, USA). They were then transformed into competent *E. coli* JM109. A total of 130 white clones for each fecal sample were randomly selected for enrichment. The purified plasmid DNA was used for sequence analysis. To verify the repeatability, we repeated the 16S rRNA gene analysis of feces at admission for nine children with diarrhea of unknown etiology. The 16S rRNA gene sequences were analyzed for chimeric constructs using the Chimera Check program within the Ribosomal Database Project.

Species-level identification was performed using a 16S rRNA gene sequence similarity of ≥99% compared with the prototype strain sequence in the GenBank. Identification at the genus level was defined as a 16S rRNA gene sequence similarity of ≥97% with that of the prototype strain sequence in the GenBank, and the sequences were listed by genus. The sequences matched attributable to either *E. coli* or *Shigella* sp. were listed as *E. coli/Shigella* sp.

### Isolation of suggested fecal-dominant *Streptococcus*

Strains of *Streptococcus* were isolated from fecal samples using KF Streptococcus Agar(Oxiod, Hampshire, United Kingdom), and identified using the MicroScan WalkAway SI 40 system(Dade Behring,West Sacramento, CA, USA). The full length of the 16S rRNA gene sequence was obtained for confirmation of identification. Pulsed-field gel electrophoresis was performed according to the protocol for *Streptococcus suis*[12]. The DNA was digested with 40 U *Sma*I (TaKaRa, Dalian, China). A dendrogram of isolates was drawn using BioNumerics software (version 4.0, Applied Maths BVBA, Belgium). Clustering of patterns was performed using the unweighted pair group with arithmetic averaging (UPGMA).

### Genome sequencing and analysis of *Streptococcus lutetiensis*

The genome of *S. lutetiensis* 033 isolated from Patient 033 was sequenced using a combination of 454 sequencings with a Roche 454 FLX and paired end sequencing derived from the pUC18 library using an ABI 3730 Automated DNA Analyzer (Applied Biosystems, Foster City, CA, USA). The genome was predicted using Glimmer software [13]. All putative open reading frames (ORFs) were annotated using non-redundant nucleotides and proteins in the NCBI, Swissport and KEGG databases. BLASTN and Artemis Comparison Tool (ACT) were used for the pair alignment. Orthologous gene clusters were searched for using the orthoMCL pipeline. We clustered these orthologous genes according to their presence or absence in different genome sequences among *Streptococcus* spp., and then a phylogenic tree was constructed using the neighbor-joining method. Genome islands were defined as having abnormal GC content with at least five continuous genes. The homologous genes within each island were compared with the references using BLASTN with an e-value cutoff at 1×10^–5^.

### Nucleotide sequence accession numbers

The GenBank accession numbers reported in this study are CP003025 for the genome sequence of *S. lutetiensis* strain 033; and JN581988 and JN581989 for the 16S rRNA gene sequences of *S. gallolyticus* subsp. *pasteurianus* strains 017 and 035, respectively.

### Ethics statement

Feces samples were acquired with the written informed consent from the parents of the children with diarrhea and normal children. This study was reviewed and approved by the ethics committee of the National Institute for Communicable Disease Control and Prevention, China CDC, according to the medical research regulations of the Ministry of Health, China (permit number 2006-16-3).

## Results

### Detection of enteric pathogens in feces of children with diarrhea

**Figure 1:**
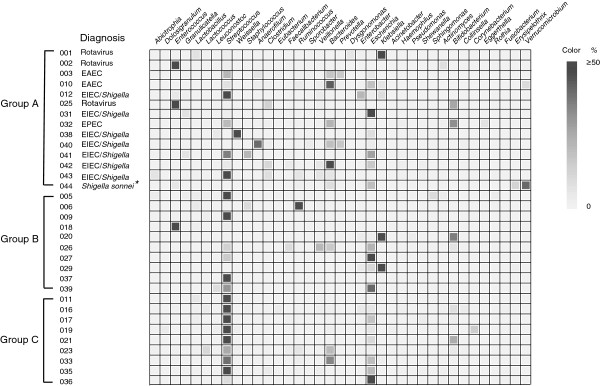
**Microbiota in the feces of children with diarrhea at admission.** Each block represents a bacterial genus. The color value changes from red to yellow and displays the percentage value (50% to 0%) of a given bacterial genus. The bacterial genera with fewer than five determined sequences, or <1% in a given sample, or unrecognized bacteria are not shown. The patients were divided into three groups. Group A were patients with diarrheagenic *E. coli* and *Shigella*. Group B were patients with diarrhea of unknown etiology and fecal samples collected only at admission. Group C were patients with diarrhea of unknown etiology and fecal samples collected at admission, during recovery, and after recovery. *S. sonnei was isolated from patient 044.

From August 17 to 30, 2006, fecal samples were obtained from 33 children with diarrhea admitted to the Children’s Hospital, Shanxi Province, China (Additional file 1: Table S1). Thirty-two of 33 children with diarrhea yielded negative culture for common enteric bacterial pathogens, such as *Salmonella*, *Vibrio* or diarrheagenic *E. coli*. *Shigella sonnei* was isolated from one patient (Figure 1). The 16S rRNA gene sequences of fecal samples were also negative for *Salmonella*, *Vibrio* or *Yersinia* spp. Eleven children with diarrhea were diagnosed with *Shigella* or diarrheagenic *E. coli*, including two with EAEC, one with EPEC, and eight with EIEC/*Shigella*, according to virulence gene detection results (Figure 1). These 11 children belonged to a group of 26 who had the 16S rRNA gene sequence of *E. coli/Shigella* sp.

The 16S rRNA gene sequence of *Bacteroides fragilis* was detected in five children with diarrhea, but its virulence gene heat-labile protein toxin was not detected. Twelve of 33 children with diarrhea were positive for the *Clostridium* 16S rRNA gene sequence, but the virulence gene toxin A or B of *Clostridium difficile* was not detected. Three samples were positive for group A rotavirus by ELISA and none tested positive for HuCV, Adv and HastV (Figure 1).

### Dominant fecal bacteria in children with diarrhea of unknown etiology

We divided the 33 children with diarrhea into three groups based on the etiological diagnosis. Group A included 14 children who were infected with diarrheagenic *E. coli* or *Shigella* species and rotaviruses. Group B included 10 children with diarrhea of unknown etiology with only one fecal sample collected at admission. Group C included nine children with diarrhea of unknown etiology from whom three fecal samples were collected, including one at admission, one during recovery, and one after recovery (Figure 1). The 16S rRNA gene sequencing data revealed that 11 of 19 children with diarrhea of unknown etiology had *Streptococcus* as the dominant fecal bacterial genus at admission. Among the remaining eight children, *Escherichia* (*n =* 4), *Klebsiella* (*n =* 2), *Enterococcus* (*n =* 1) or *Ruminococcus* (*n =* 1) was the most dominant bacterial genus (Figure 1).

We analyzed fecal samples from five healthy and five hospitalized children at the same location but with no apparent diarrhea as controls. None of the genera *Escherichia*, *Enterococcus*, *Klebsiella*, *Ruminococcus* and *Streptococcus* was dominant within the control fecal samples taken from five healthy children. None of five hospitalized children at the same location but with no apparent diarrhea had *Streptococcus* as the dominant genus, although one of them had the percent of *Streptococcus* to 34.96% in fecal microbiota. There were no species in the *Streptococcus bovis* group in the 10 children in the control group (Additional file 2: Figure S1).

**Figure 2:**
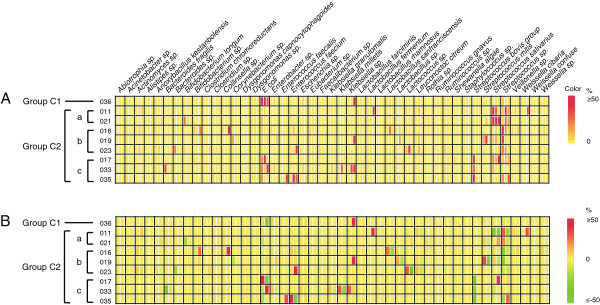
**Percentage changes in fecal bacteria in children with diarrhea at admission and during and after recovery.** Only patients who had unknown etiology and provided three fecal samples were included. The bacterial species with fewer than five determined sequences, or <1% in a given sample, or unrecognized species are not shown. (**A**) The true percentage value of individual bacterial species in fecal samples of patients with diarrhea sampled at admission and during and after recovery. Each block was divided into three columns by white vertical lines, representing the fecal samples at admission and during and after recovery, respectively. The color value from red to yellow displayed the percentage (50% to 0%) of a given bacterial species in each sample. (**B**) The percentage changes in individual bacterial species in fecal samples from patients with diarrhea during and after recovery compared with that at admission. Each block was divided into two columns by white vertical lines, representing the relative percentage changes of given bacterial species during and after recovery, compared with that in feces at admission. The color value from red to yellow to green displayed the percentage (50% to 0% to −50%) of a given bacterial species in each sample. The negative percentage shows that the percentage of a given bacterial species was reduced compared with that detected at admission.

In nine children with diarrhea of unknown etiology in Group C, eight had *Streptococcus* as the most dominant fecal bacterial genus at admission, one with *S. lutetiensis*, two with *S. gallolyticus* subsp. *pasteurianus*, two with *Streptococcus salivarius*, and three with *Streptococcus* sp. (Figures 1 and 2, Table 1). We divided these nine children in Group C into two, according to the most dominant fecal bacterial species at admission. Group C1 included one child whose most dominant species was *E. coli*. The percentage of *E. coli* in the fecal microflora of Patient 036 (age 7 months) was increased from 87.10% at admission to 90.91% during treatment, and then dropped to 28.90% after recovery (Figure 2B), based on 445 analyzed 16 s rRNA gene sequences.

**Table 1:** Features of study samples from children with diarrhea of unknown etiology

**Patient information**		**Clinical presentation**			**Stool routine analysis**		
**Patient and feces number**	**Sampling date (after onset)**	**Times of stool/day**	**Characteristics of stool**	**Temperature (°C)**	WBC*	RBC*	**Occult blood**
011-1	1	5	Watery	Normal	+	++	+
011-3	3	5	Loose				
011-4	5	2	Formed				
016-1	1	3-4	Bloody and mucoid	39.0°C	++	+	+/−
016-3	3	3	Watery				
016-6 ^**^	12	2	Formed				
017-1	16	10	Watery	Normal	+	++	+/−
017-3	18	6	Watery				
017-5	20	6	Watery				
019-1	133	8-9	Bloody and mucoid	Normal	++	++	+
019-6	138	3	Loose				
019-7^**^	143	3	Loose				
021-1	33	6	Watery	Normal	+	+	-
021-4	35	5	Watery				
021-7	38	4-5	Loose				
023-1	20	6	Loose	38.7°C	++	-	-
023-5	24	3	Formed				
023-6^**^	28	2-3	Formed				
033-1	5	5	Watery	Normal	-	-	-
033-3	7	2	Formed				
033-5	9	2	Formed				
035-1	1	5	Bloody and mucoid	38.3°C	+	++	+
035-4	4	2-3	Formed				
035-6 ^**^	9	2-3	Formed				
036-1	7	6	Loose	Normal	++	+	+
036-2	8	3	Loose				
036-3	9	2	Loose				

* +: 6–10/ high power field (HPF) ++: >10/HPF.

**: Fecal samples collected at patient discharge from hospital.

Group C2 included eight children with diarrhea, who were further divided into three subgroups, based on the most dominant fecal bacterial species at admission. Group C2a included two children who had *S. salivarius* as the most dominant fecal bacterial species. Group C2b included three children who had *Streptococcus* sp. as the most dominant species. Group C2c included three children who had *S. bovis* group as the most dominant species (Figure 2A and B).

For Patient 011 (age 2.5 years) in Group C2a, the percentage of *S. salivarius* in the fecal microflora was reduced from 78.95% at admission to 31.43% during recovery (Figure 2B), based on 442 sequences analyzed. Patient 021 (age 8 months) had the percentage of *S. salivarius* in the fecal microflora of 58.56% at admission, which increased to 60.0% during recovery and then to 76.67% after recovery (Figure 2B).

Group C2b had *Streptococcus* sp. as the dominant fecal species at admission. For Patient 016 (age 9 months), the percentage of *Streptococcus* sp. in fecal microflora was reduced from 51.28% to 15.65% during recovery (3 days of treatment), and then to 4.67% after recovery (12 days of treatment) (Figure 2B), based on 456 16S rRNA gene sequences analyzed. For Patient 019 (age 4 months), the percentage of *Streptococcus* sp. in fecal microflora was reduced from 40.54% at admission to 7.08% during recovery (6 days of treatment) and then to 1.77% after recovery (11 days of treatment) (Figure 2A and B), based on 448 16S rRNA gene sequences analyzed. For Patient 023 (age 5 months), the percentage of *Streptococcus* sp. in fecal microflora was reduced from 26.05% at admission to 13.56% during recovery (5 days of treatment) and then to zero after recovery (9 days of treatment) (Figure 2B), based on 440 16S rRNA gene sequences analyzed.

All three patients in Group C2c had *S. bovis* group as their most dominant fecal bacterial species at admission. For Patient 033 (age 2 months), the percentage of *S. bovis* group in fecal microflora was reduced from 26.84% at admission to zero during recovery (3 days of treatment) (Figure 2B). It was not detected in feces sampled at discharge from the hospital, after 5 days of treatment. For Patient 017 (age 1.5 years), the percentage of *S. bovis* group in fecal microflora was reduced from 39.82% at admission to zero during recovery (3 days of treatment) (Figure 2B). It was not detected in feces sampled at discharge from hospital, after 5 days of treatment. For Patient 035 (age 8 months), the percentage of *S. bovis* group in fecal microflora was reduced from 42.73% at admission to zero after 4 days of treatment (Figure 2B). It was not detected in the feces sampled at discharge from hospital, after 9 days of treatment.

### Isolation and identification of the *S. bovis* group from feces

We attempted to culture the dominant bacterial species as identified by the 16S rRNA gene analysis from the feces of all nine patients in Group C (Figures 1 and 2). Four patients (016, 019, 021 and 023) had negative cultures even on non-selective blood agar; possibly because antibiotics had been given before the hospital consultation.

**Figure 3:**
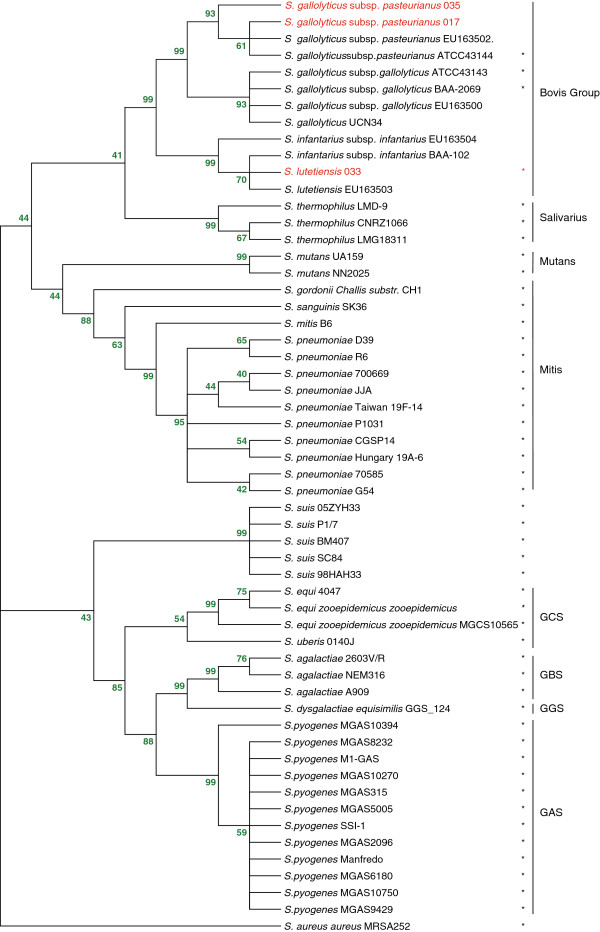
**Phylogenetic analysis of isolated strains of the *S. bovis *group and other major streptococcal species based on complete 16S rRNA gene sequences.** The multiple sequence alignment of 16S rRNA genes was performed using ClustalW. The conserved tree was constructed using the neighbor-joining method. Bootstrap values are shown above each branch. All 16S rRNA gene sequences were derived from the NCBI and validated using genome sequences. The strains with complete genomes are marked with a star to the right of the species name. *Staphylococcus aureus* subsp. *aureus* MRSA252 was included as an out-group. The strains in red were isolated in this study.

Patient 017 had seven isolates belonging to the *S. bovis* group in the feces samples collected at admission, Patient 033 had 19, and Patient 035 had 10. According to the results of the MicroScan WalkAway SI 40 system, all isolates of the *S. bovis* group were identified as biotype II (mannitol fermentation negative). We then amplified, cloned, and sequenced the major portion of the 16S rRNA gene from each isolate. The strains isolated from Patient 033 were identified as *S. lutetiensis* and those from Patients 017 and 035 were *S. gallolyticus* subsp. *pasteurianus.* A dendrogram comparing representative 16S rRNA gene sequences of the isolated *S. bovis* group strains with other *Streptococcus* species mapped our isolates within the *S. bovis* group (Figure 3).

Chromosomal DNA from the 36 strains of the *S. bovis* group from the three patients were digested with restriction enzyme *Sma*I and analyzed using pulsed-field gel electrophoresis (PFGE). Strains from each patient (seven from Patient 017, 19 from Patient 033 and 10 from Patient 035) were found to have unique restriction patterns.

### Genome sequence and comparison of the *S. bovis* group with *S. lutetiensis***strain 033**

**Figure 4:**
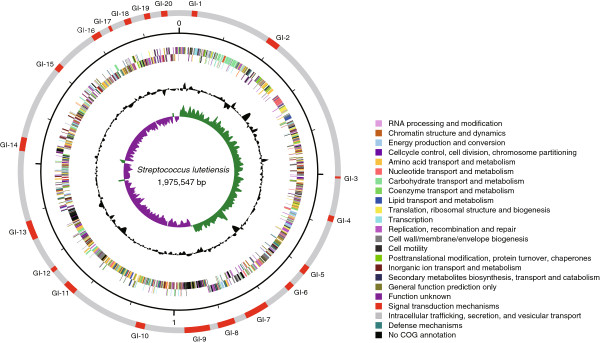
**Genome sequence of *S. lutetiensis *strain 033.** Key to the circular diagram (outer to inner): (1) GI found in the chromosome. (2) *S. lutetiensis* strain 033 COG categories on the forward strand (+) and the reverse strand (−). (3) G + C content and GC skew (G-C/G + C) of 033, respectively, with a window size of 10 kb.

We sequenced the entire genome of the *S. lutetiensis* strain 033 and compared it withits close relatives, *S. gallolyticus* subsp. *pasteurianus* and *S. gallolyticus* subsp. *gallolyticus *[14]. To the best of our knowledge, this is the first time the genome of *S. lutetiensis* has been completely sequenced. The genome of strain 033 contained 1,975,547 bp with a GC content of 37.7%. It had 60 tRNAs and 18 rRNAs (six operons). Fifty-five tandem repeated regions were identified in the genome with the highest number of tandem repeats duplicated 104 times (at 3,744 bp, genome position from 844,798 to 848,542). A total of 2,015 ORFs >300 bp (100 aa) were identified. Of these, 86.2% matched clusters of orthologous groups (COGs) in the database with e-values <1×10^**–5**^ (Figure 4).

**Figure 5:**
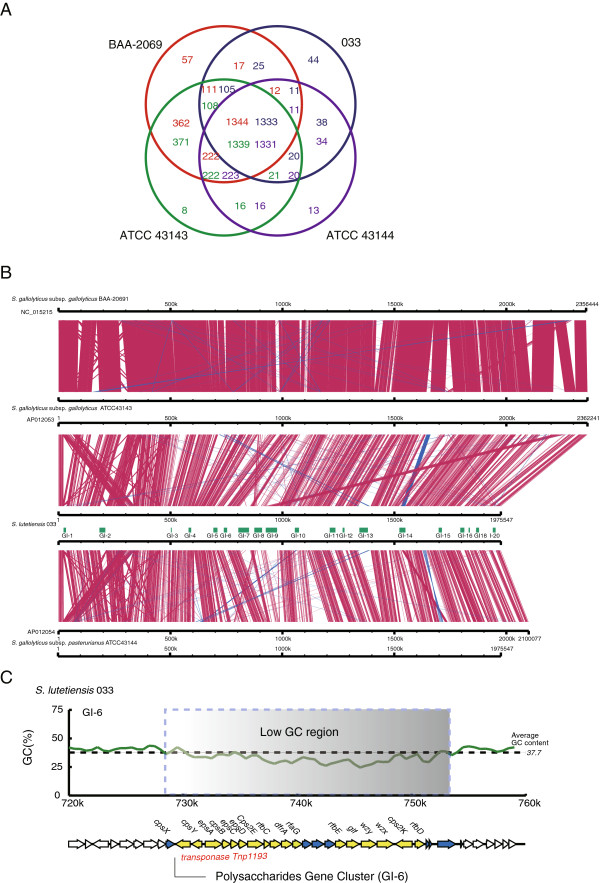
**Genome analysis of *S. lutetiensis *strain 033.** Comparative analysis of all completed genomes of the *S. bovis* group (*S. gallolyticus* subsp*. gallolyticus* BAA-2069, *S. gallolyticus* subsp. *gallolyticus* ATCC43143, and *S. gallolyticus* subsp. *pasterurianus* ATCC43144). (**A**) Venn diagram of homologous genes in four complete genomes. The number of homologous genes is noted in each circle: red for BAA-2069, blue for 033, green for ATCC43143, and purple for ATCC43144. (**B**) Local collinear block of the chromosome sequences of four genomes. The red blocks represent similar regions within nucleotide sequences, and the blue block represents a region similar to the complementary strands. GIs in our 033 genome are shown in the green block near the genome. (**C**) Organization of GI-6 encoding CPS. GC contents calculated using each 1 kb with a 500-bp step. The direction of the arrows represents the coding strand of the ORFs. The genes in the GIs are marked with blue (unknown functions) and yellow (known functions).

Twenty genomic islands (GIs) in the genome of *S. lutetiensis* 033 were identified. Of these, five were antibiotic-resistance islands and two were putative pathogenicity islands (Figure 4). Notably, GI-7 was found to contain four glycosyl transferase genes, four pilin-related genes, and >10 transposase genes or putative transposase genes that have been reported to be associated with virulence in *Streptococcus pneumoniae****,****Neisseriaceae*, and others [15-17]. GI-18 encodes a colonization-associated adhesion factor previously described in *S. suis*[18]. GI-6 encodes the capsule polysaccharide (CPS) genes that are associated with the virulence of pathogenic streptococci; for example, *S. pneumoniae* and *S. suis* (Figure 5C) [19-21]. Five GIs were unique to *S. lutetiensis* and have not been identified in other species of this genus. Two were phage related, one encoded a cellobiose phosphorylase-like protein, one encoded an ATPase, and one had an unknown function. We found the hemolytic toxin *cylZ* in *S. lutetiensis* that activates the neutrophil signaling pathways in the brain endothelium and contributes to the development of meningitis identified in *S. agalactiae*[22]. The gene for sortase (*SrtA*), also identified in the genome of *S. lutetiensis*, was found to be associated with adhesion to epithelial cells and with colonization of pathogenic streptococci [23-25] (Table 2).

**Table 2:** **Putative virulence genes detected in the genome of *S. lutetiensis *strain 033**

**Virulence factors**	**Genes related**	**Putative function**	**References**
Pneumcoccal cell surface adherence protein A	*pavA*	Fibronectin binding	[26]
Laminin-binding protein	*lmb*	Colonization	[22]
Pilus-associated adhesin	*rrgA*	Colonization	[18]
Sortase A	*srtA*	Adhesion to epithelial cells	[27]
Streptococcal lipoprotein rotamase A	*slrA*	Colonization	[28]
Streptococcal enolase	*eno*	Plasminogen binding	[29]
Pneumococcal surface antigen	*psaA*	Adhesin	[30]
C3-degrading protease	*cppA*	Evasion of innate immunity	[31]
Serine protease	*htrA/degP*	Biogenesis of Streptolysin S	[14]
Trigger factor	*tig/ropA*	Stress tolerance and biofilm formation	[32]

Together with the biochemical tests, the results of 16S rRNA gene sequencing with 48 complete genomes for streptococci, two draft genomes (*Streptococcus infantarius* subsp. *infantarius* BAA-102 and *S. gallolyticus* UCN34), and four segment 16S rRNA genes (EU163500, EU163502, EU163503, and EU163504) in the *S. bovis* group were selected for an evolutionary study. The reference strain of *S. lutetiensis* (accession number: EU163503) was found to be the nearest strain to the *S. lutetiensis* genome sequence in our study, showing the same 16S rRNA gene sequences. Compared with the nearest species *S. infantarius* subsp. *infantarius* BAA-102 and EU163504, strain 033 had two and three nucleotide differences in the 16S rRNA genes, respectively.

An entire genome comparative analysis was performed on the four completed genomes of *S. gallolyticus* subsp*. gallolyticus* BAA-2069, *S. gallolyticus* subsp. *gallolyticus* ATCC43143, and *S. gallolyticus* subsp. *pasterurianus* ATCC43144 in the *S. bovis* group. The *S. lutetiensis* sequenced genome in our study was found to be phylogenetically related to the genome of *S. gallolyticus* subsp. *pasterurianus* ATCC43144; and 94.1% of the genes were found in the homologous genes in ATCC43144 (Figure 5A) [14]. Although large-scale genome rearrangements, inversions and deletions were observed, the four genomes displayed the same collinear structure (Figure 5B). We found 15.2% of the genes of *S. gallolyticus* subsp. *pasterurianus* and 34.9% of the genes of *S. gallolyticus* subsp. *gallolyticus* were not present in *S. lutetiensis*, suggesting that the genome of *S. lutetiensis* strain 033 was similar to that of *S. gallolyticus* subsp. *pasterurianus* (Figure 5A).

## Discussion

Selective media are routinely used to isolate particular pathogens from mixtures of bacterial species from the feces of patients with diarrhea. However, they cannot be used to isolate putative bacterial agents of diarrhea of unknown etiology. The important feature of the direct sequencing of the 16S rRNA gene in the fecal samples is the ability to identify most of the existing bacterial species [33]. Using this technique, we analyzed the dynamics of the fecal bacteria flora in nine patients with diarrhea of unknown etiology. We examined three fecal samples per patient, at admission, during recovery, and after recovery. We speculated that the putative causative enteric pathogens were dominant in the intestine when infection was established. The number of causative pathogens in the intestine may decrease during treatment and after recovery.

Eight of nine patients (Group C2) who provided all three specimens with unknown etiology at admission had as the dominant *Streptococcus* genus in their fecal samples. There is a report of a child who developed hemolytic uremic syndrome with group A beta hemolytic *streptococcus*-positive diarrhea [34]. Streptococci are also numerous in the fecal microflora of patients with irritable bowel syndrome patients [35]. So, the role of streptococci in the fecal microflora of children with diarrhea deserved further research.

Three patients from Group C2 had *Streptococcus* as the dominant genus, and all showed a reduced the percentage of *Streptococcus* sp. in fecal microflora of during and after recovery. Two patients had *S. salivarius* as the dominant species with one showing a reduced the percentage of *Streptococcus* sp. in fecal microflora during and after recovery. The other patient showed an increase. Three patients had the *S. bovis* group as the dominant species, and all showed a reduced the percentage of *S. bovis* group in fecal microflora during and after recovery. This observation suggests that the association of the *S. bovis* group with diarrhea is worthy of further investigation.

*S. bovis* is divided into three biotypes, I (*S. gallolyticus* subsp. *gallolyticus*), II/1 (*S. lutetiensis* and *S. infantarius*), and II/2 (*S. gallolyticus* subsp. *pasteurianus*), based upon mannitol fermentation and β-glucuronidase activities. *S. gallolyticus* subsp. *gallolyticus* is known to be associated with endocarditis and colon carcinoma. *S. infantarius*, *S. lutetiensis* and *S. gallolyticus* subsp. *pasteurianus* are associated with non-colonic cancer and meningitis. Children with signs of gastrointestinal disturbance at presentation associated with *S. bovis* were also reported [36].

The dominant species from the nine patients of group C were cultured and four showed that they were negative. Thirty-six strains of the *S. bovis* group were isolated from three patients, and PFGE analysis showed that they had their own unique restriction pattern, indicating that the strains within individual patients were identical. The isolates were identified as *S. lutetiensis* and *S. gallolyticus* subsp. *pasteurianus*.

We determined and analyzed the full genome sequence of the *S. lutetiensis* strain isolated from a child with diarrhea. Two previously recognized pathogenicity islands were identified in the genome. GI-6 was found to encode a CPS gene cluster involved in the pathogenicity of *S. suis*[21]. GI-7 was found to encode glycosyl transferase, the virulence factor in *S. pneumoniae*[17]. Eight additional virulence factors were identified in the *S. bovis* group. These included the putative hemolytic toxin *cylZ* and the sortase gene associated with adhesion and colonization [22,24,25].

## Conclusions

We studied the dynamics of the fecal microbial community in children with diarrhea of unknown etiology and found for the first time that strains of the *S. bovis* group were among the predominant bacteria in some of the patients at admission, and showed a reduction in numbers during treatment and recovery. In addition, we report the first genome sequence of a *S. lutetiensis* isolate, identifying putative pathogenic islands and virulence genes. However, it was hard to detect all the infectious agents and there were many non-infectious factors that may cause diarrhea; therefore, additional studies are needed to clarify the potential contribution of these bacteria to diarrhea in children.

## Competing interests

The authors declare that they have no competing interests.

## Authors’ contribution

DJ, LL, ZL, HJ, CY and JX conceived and designed the experiments.DJ, SL,YXu and XB performed the experiments.CC, ZZ, PD, HW, YXiong, HZ and LW carried out the molecular genetic studies and participated in the sequence alignment.HS contributed reagents and materials. DJ, MG and JX wrote the manuscript. All authors read and approved the final manuscript.

## Acknowledgements

This work was supported by grants (2011CB504901, 2008ZX10004-001, 2008ZX10004-009, 2009ZX10004-101, 2011SKLID209) from the Ministry of Science and Technology, the National Key Programs for Infectious Diseases of China; and by grants from the State Key Laboratory for Infectious Disease Prevention and Control, People’s Republic of China.

## Supplementary Material

Additional file 1: Table S1 Characteristics of patients and clinical presentation of diarrhea among children included in this study. Click here for file

Additional file 2: Figure S1 Dominant bacterial species in the feces of the control group. Click here for file
