# Supplementary material for: Dynamics of fecal microbial communities in children with diarrhea of unknown etiology and genomic analysis of associated Streptococcus lutetiensis
Source: BMC Microbiol. 2013 Jun 19;13:141. doi: 10.1186/1471-2180-13-141 (PMC3716944; doi:10.1186/1471-2180-13-141)
Supplement: Additional file 1: Table S1 — Characteristics of patients and clinical presentation of diarrhea among children included in this study. [file 1471-2180-13-141-S1.doc]

**Supplemental Table: Characteristics of patients and clinical presentation of diarrhea among children included in this study.**

| **Patient information** | | **Clinical presentation** | | | **Stool routine analysis** | | |
| --- | --- | --- | --- | --- | --- | --- | --- |
| **Patient**  **Number** | **Sampling**  **Date(after onset)** | **Times of stool /day** | **Characteristics of stool** | **Temperature**  **(℃)** | **WBC*** | **RBC*** | **Occult blood** |
| 001 | 7 | 4-5 | Loose | Normal | - | - | - |
| 002 | 10 | >10 | Bloody and mucoid | 40.5℃ | ++ | + | + |
| 003 | 3 | 5-6 | Watery | 37.8℃ | - | - | - |
| 005 | 7 | 3-4 | Loose | Normal | - | - | - |
| 006 | NA** | 8-9 | Watery | Normal | - | - | - |
| 009 | 33 | 5-6 | Watery | 38.7℃ | + | + | + |
| 010 | 0 | 8 | Loose | Normal | - | - | - |
| 011 | 1 | 5 | Watery | Normal | + | ++ | + |
| 012 | 1 | 7-8 | Bloody and mucoid | 39.0℃ | ++ | + | + |
| 016 | 1 | 3-4 | Bloody and mucoid | 39.0℃ | ++ | + | +/- |
| 017 | 16 | 10 | Watery | Normal | + | ++ | +/- |
| 018 | 1 | 6 | Loose | 38.9℃ | ++ | - | - |
| 019 | 133 | 8-9 | Bloody and mucoid | Normal | ++ | ++ | + |
| 020 | NA** | 10 | Watery | Normal | - | - | - |
| 021 | 33 | 6 | Watery | Normal | + | + | - |
| 023 | 20 | 6 | Loose | 38.7℃ | ++ | - | - |
| 025 | NA** | 7 | Bloody and mucoid | 39.5℃ | - | - | + |
| 026 | 15 | 7 | Watery | 37.9℃ | - | - | - |
| 027 | 9 | 8 | Watery | 37.8℃ | - | - | - |
| 029 | 7 | 5 | Watery | Normal | - | - | - |
| 031 | 1 | 6 | Watery | 38.8℃ | 3+ | + | + |
| 032 | 3 | 4 | Watery | Fever | - | - | - |
| 033 | 5 | 5 | Watery | Normal | - | - | - |
| 035 | 1 | 5 | Bloody and mucoid | 38.3℃ | + | ++ | + |
| 036 | 7 | 6 | Loose | Normal | ++ | + | + |
| 037 | 7 | 6 | Loose | Fever | 3+ | + | + |
| 038 | 1 | 6 | Bloody and mucoid | 39.2℃ | 3+ | + | + |
| 039 | 153 | 10 | Watery | Normal | - | - | - |
| 040 | 3 | 4 | Loose | 40℃ | + | - | - |
| 041 | 0 | 4 | Bloody and mucoid | 38.5℃ | ++ | + | + |
| 042 | 1 | 10 | Watery and bloody | 37.2℃ | 3+ | 3+ | + |
| 043 | 3 | 6 | Bloody and mucoid | 39.5℃ | ++ | ++ | + |
| 044*** | 1 | 10 | Watery | 37.8℃ | ++ | + | + |

* +: 6–l0/high power field (HPF) ++: >l0/ HPF; 3+: too numerous to count

** Not applicable

*** Isolated *S. sonnei* using routine isolation methods
